# Scanning double-sided documents without incurring show-through by learning to fuse two complementary images using multilayer perceptron

**DOI:** 10.1371/journal.pone.0176969

**Published:** 2017-05-10

**Authors:** Yuzhong Chen

**Affiliations:** School of Material Science and Engineering, Shandong University, Jinan, China; Tianjin University, CHINA

## Abstract

This paper presents a novel method for scanning duplex-printed documents without incurring the unwanted show-through artifact. The proposed method achieves the goal of eliminating the leaked-out reverse-side content by fusing a white backed scan image with a black backed scan image of the document. The fusion is accomplished using a multilayer perceptron having learned a fusion mapping from manually corrected document images. The main novel contributions of this work include (1) being the first to propose to accomplish the goal of show through free scanning by fusing a white backed scan image with a black backed scan image of the document; (2) proposing a learning approach using a multilayer perceptron to learn the fusion mapping from manually corrected scan images; and (3) proposing to use the pixel value histogram of reverse-side-printed area as well as the pixel value histogram of duplex-printed area to quantitatively indicate show through severity to facilitate objective comparison of the methods in consideration. The experiment results show that the proposed method is remarkably more powerful in eliminating show through than the two state-of-the-art methods in comparison.

## Introduction

**Fig 1:**
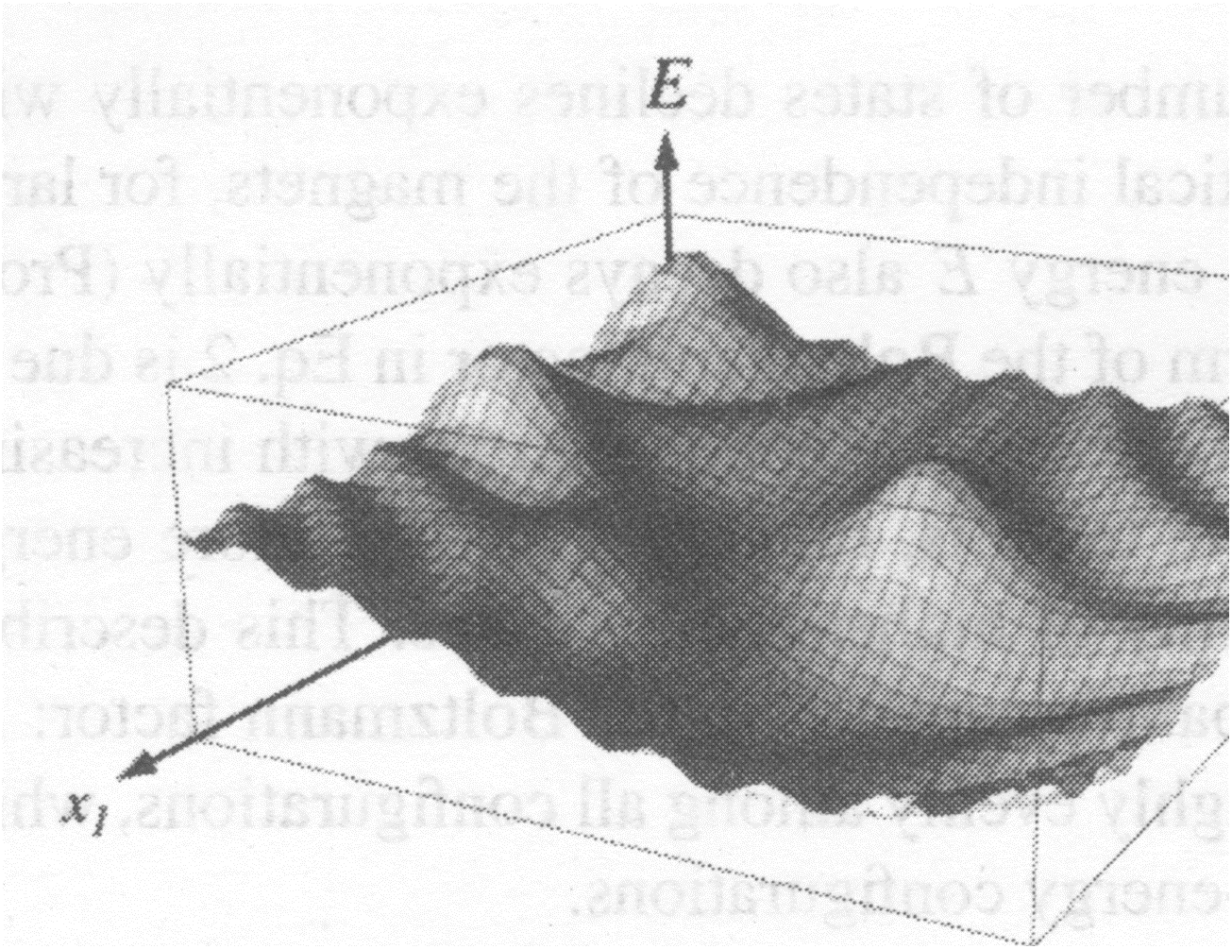
Part of the scan image of a duplex-printed book page.

**Fig 2:**
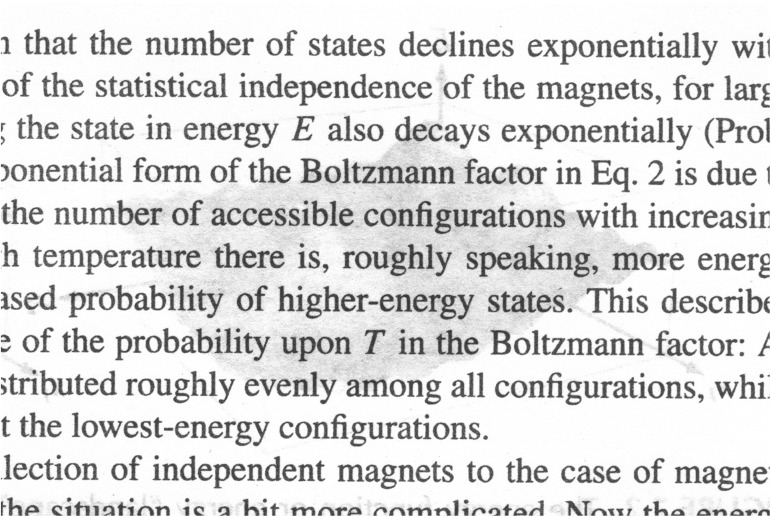
Part of the scan image of the reverse side of the page displayed in Fig 1.

Document scanning has become an office routine being performed every day and everywhere to capture digital image of document page for convenient storage, copying, transmission, processing, analysis, and recognition etc. One major deficiency of the existing scanning methods is that the text and image content on the reverse side of duplex-printed document may show through the paper substrate to appear in the scan image. Fig 1 displays a part of the scan image of a duplex-printed book page where the reverse side content (displayed in Fig 2) leaks out into the scan image. The lower the paper substrate’s opacity, the severer the show through becomes. The leaked out reverse side text and image content reduces the scan image’s aesthetic quality, decreasing its readability for both the human eye and the Optical Character Recognition (OCR) system.

Considerable research has been performed over the past decades to develop methods to eliminate the unwanted show through artifact. One existing approach is to apply a sheet of black paper to back the document for scanning [1]. This method significantly suppresses show through thanks to the reduction of the light reflected back to the page from the backing surface. This approach however brings about the undesirable side effects of showing up the material texture of the paper substrate, and producing black spots if the document contains worn out holes, and giving rise to black borders if the document is smaller than the scan area.

Another existing way to overcome the show through problem is to scan both the front side and back side of the page, and then use the front side image and a flipped and registered version of the reverse side image to achieve the goal [2–9]. This class of methods does not produce the side effects of [1], but they require accurate registration of the two scan images which is often difficult to accomplish since the leaked out reverse side content is typically blurred and much fainter than the front side content resulting in registration difficulty and error that will significantly decrease the performance and may even lead to failure of these methods. The performance of this group of methods also relies on accurate modeling of the show though optical process which is difficult to obtain, resulting again in performance degradation.

As of today, the performance of the existing methods have not been adequate, and the research problem of how to better overcome the show-through interference is still open. Our work is motivated from a new insight, that is, from the observation that the advantages of applying white backing to the document during scanning are complementary to that of applying black backing. Inspired by this insight, we have developed a method to fuse the two images to take both advantages. The experiment results show that our proposed approach offers significantly better performance than the state-of-the-art methods in comparison.

The main novel contributions of this work include (1) being the first to use a learned fusion mapping to fuse a white backed scan image with a black backed scan image of the document to realize the goal of show through free scanning; (2) proposing a learning approach using a multilayer perceptron to learn the fusion mapping from manually corrected scan images; and (3) proposing to use the pixel value histogram of reverse-side-printed area as well as the pixel value histogram of duplex-printed area as a measure of show through severity to facilitate objective comparison of the methods in consideration.

The remaining part of this paper is organized to first present the proposed method in the next section, and then to present and discuss the experiment results in the subsequent section, and finally to draw conclusions in the last section.

## The proposed show-through elimination method

**Fig 3:**
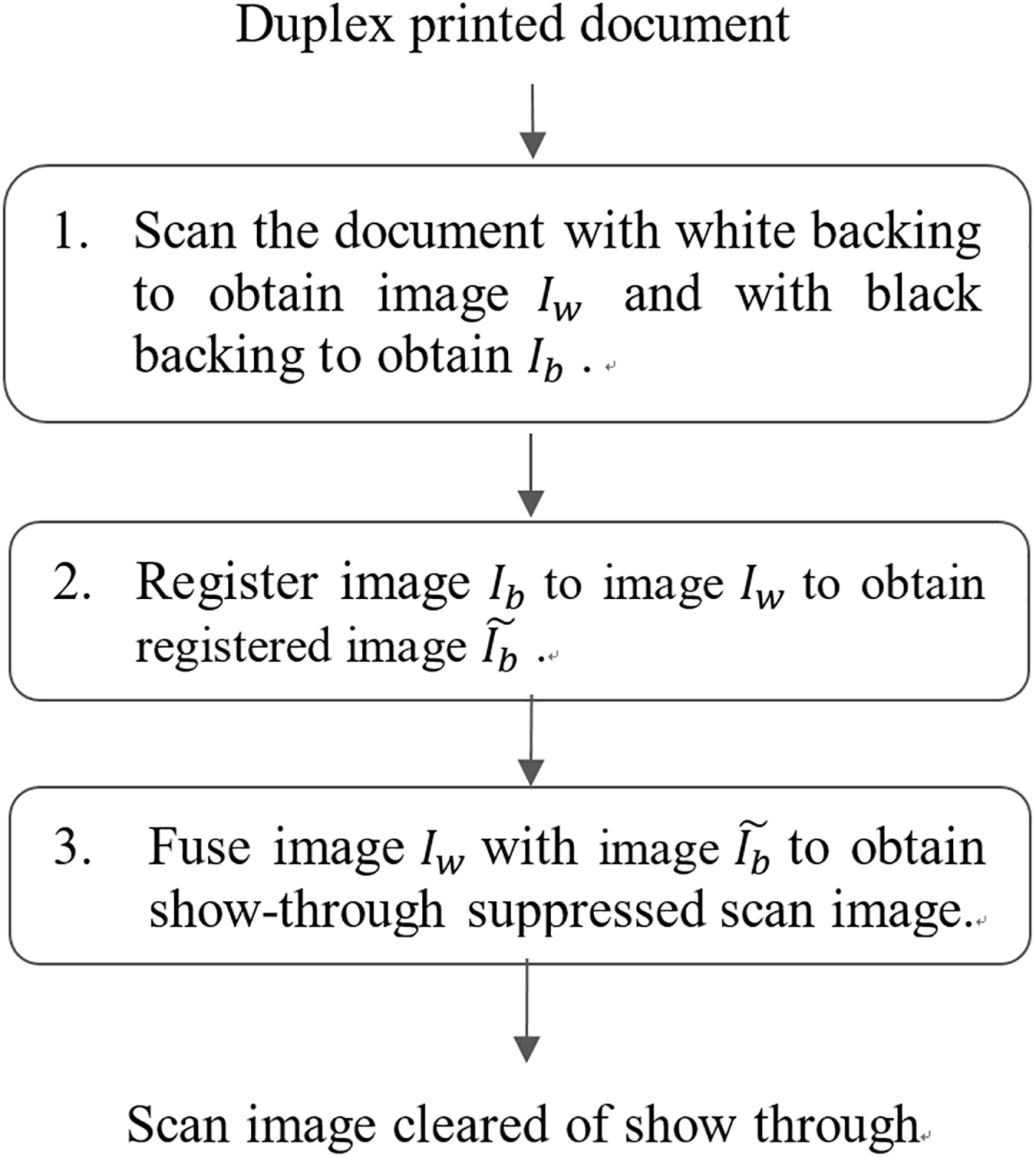
Work flow of the proposed method.

Inspired by the observation that applying black backing to a document for scanning offers advantages complementary to applying white backing, we have developed a method to fuse the two scan images using a multilayer perceptron to achieve the goal of show through free scanning of two-sided documents. The proposed method is comprised of three steps as shown in Fig 3.

### Capturing white backed scan image and black backed scan image

The first step of the proposed method is to use the backing surface of the scanner which is usually white (otherwise to use a sheet of white paper) to back the document and scan it to obtain a white backed scan image I_w_, and then use a sheet of black paper to back it to obtain a black backed scan image I_b_.

### Registering black backed scan image to white backed scan image

The second step is to register black backed scan image I_b_ to white backed scan image I_w_ in order to be able to fuse them. To this aim, we first detect key points in images I_b_ and I_w_ using Scale Invariant Feature Transform (SIFT) [10, 11] which has been reported to be powerful and robust. Other detectors such as SURF [12] and SUSAN [13] may also be used. We then match the detected key points of the two images according to the similarity of their descriptor vectors. Thereafter we use RANSAC [14, 15] to determine the parameters (a, b, θ) of the rigid transform Ψ (Eq 1) from the I_b_ plane to the I_w_ plane

$$\left(\begin{array}{c} x^{\prime }\\ y^{\prime } \end{array}\right)=\left(\begin{array}{cc} \cos\theta & \sin\theta \\ -\sin\theta & \cos\theta \end{array}\right)\left(\begin{array}{c} x\\ y \end{array}\right)+\left(\begin{array}{c} a\\ b \end{array}\right)$$ (1)

We then apply the transform Ψ and use bilinear interpolation to obtain the registered image I_b_^~^, that is, we first compute

$$\tilde{I_{b}}(x,\lfloor \Psi_{y}\rfloor )=(1-\Psi_{x}+\lfloor \Psi_{x}\rfloor )∙I_{b}(\left\lfloor \Psi_{x}\rfloor ,\lfloor \Psi_{y}\right\rfloor )+(\Psi_{x}-\lfloor \Psi_{x}\rfloor )∙I_{b}(\left\lfloor \Psi_{x}\rfloor +1,\lfloor \Psi_{y}\right\rfloor )$$ (2)

and

$$\tilde{I_{b}}(x,\lfloor \Psi_{y}\rfloor +1)=(1-\Psi_{x}+\lfloor \Psi_{x}\rfloor )∙I_{b}(\lfloor \Psi_{x}\rfloor ,\lfloor \Psi_{y}\rfloor +1)+(\Psi_{x}-\lfloor \Psi_{x}\rfloor )∙I_{b}(\lfloor \Psi_{x}\rfloor +1,\lfloor \Psi_{y}\rfloor +1)$$ (3)

where └.┘ denotes the integer floor operator, Ψ_x_ is an abbreviated notation for the x component of Ψ(x, y).

We finally obtain

$$\tilde{I_{b}}(x,y)=(1-\Psi_{y}+\lfloor \Psi_{y}\rfloor )∙I_{b}(x,\lfloor \Psi_{y}\rfloor )+(\Psi_{y}-\lfloor \Psi_{y}\rfloor )∙I_{b}(x,\lfloor \Psi_{y}\rfloor +1)$$ (4)

### Fusing the two scan images using trained multilayer perceptron

The third step is to fuse image I_w_ with registered image I_b_^~^ using the learned fusion mapping Θ(x, y) as discussed in the next section. For monochrome image, the fusion is accomplished by

$$g(x,y)=\frac{1}{\mu }\;\vartheta \left(\mu \;I_{w}(x,y),\;\mu \;\tilde{I_{b}}(x,y)\right),\;\forall (x,y)\in I_{w}$$ (5)

in which μ is the scaling factor as discussed in Section 2.4.

For color images, the R, G, and B channels of the image are individually fused using Eq 5.

### Learning a fusion mapping with multilayer perceptron

We employ multilayer perceptron and error back propagation algorithm [16, 17] to learn the fusion mapping Θ(x, y) used in Step 3 of the proposed method (as discussed in Section 2.3). The reason for choosing multilayer perceptron and back propagation algorithm is due to its proven strong ability to learn complex mappings. Our network consists of 3 layers of sigmoidal neurons of the following sigmoidal activation function

$$y(v_{i})=\frac{1}{1+e^{-v_{i}}}$$ (6)

where y(v_i_) is the output of the i-th neuron and v_i_ is the weighted sum of the input synapses. The network has 2 inputs and 1 output, and the hidden layer contains 10 neurons.

The training samples are obtained from manually corrected scan images together with their corresponding white backed scan images and black backed scan images. For one manually corrected image I_c_, and its corresponding white backed scan image I_w_ and registered black backed scan image I_b_^~^, we obtain one training sample from each pixel triple of the image triple (I_c_, I_w_, I_b_^~^). For each training sample (p_c_, p_w_, p_b_^~^), the input to the network is (μ.p_w_, μ.p_b_^~^) and the expected output is μ.p_c_. The scaling factor μ is used to fit the pixel value range to the input and output ranges of the perceptron, and is set to 0.8/256 for conventional digital image of 8-bit-per-channel pixels.

## Experiments and discussions

We use HP Scanjet G4050 at 600 dpi resolution and duplex-printed book pages for the experiments to evaluate the performance of the proposed method and to compare it to two state-of-the-art methods [1, 9]. The proposed method is implemented in C++ with Open Source Computer Vision [18] and Fast Neural Network Library [19], and runs on an ordinary desktop PC with Intel i5-4460T CPU and 8G memory.

In addition to visual inspection and comparison of the resulting images obtained by the three methods, we propose to use the pixel value histogram of reverse-side-printed area (image area that contains reverse-side content but no front-side content) as well as the pixel value histogram of duplex-printed area (image area that contains both front-side printing and reverse-side printing) to quantitatively indicate the severity of the show-through to enable objective comparison. The pixel value histogram of reverse-side-printed area will have one highly concentrated peak at high pixel value location if the area does not contain show through, and the histogram will become less concentrated and spread more to lower pixel value region as the area contains more leaked out reverse side content. We use its standard deviation to quantitatively indicate the severity of the show-through, as a larger standard deviation value indicates more dispersion of the peak, which corresponds to severer show-through.

The learning of the fusion mapping Θ(x, y) by the multilayer perceptron is accomplished using manually corrected scan image together with the original white backed scan image and black backed scan images of one book page. The training process needs to be carried out only once and takes about 10 seconds to complete.

We use 2 duplex-printed book pages to evaluate and compare the three methods, and include the white-backed scan images and the black-backed scan images of the front side, and white-backed scan images of the reverse side of the two duplex-printed book pages in S1 through S6 Figs. We now present and analyze the experiment results of the proposed method and two state-of-the-art methods in comparison.

### Method [1]

**Fig 4:**
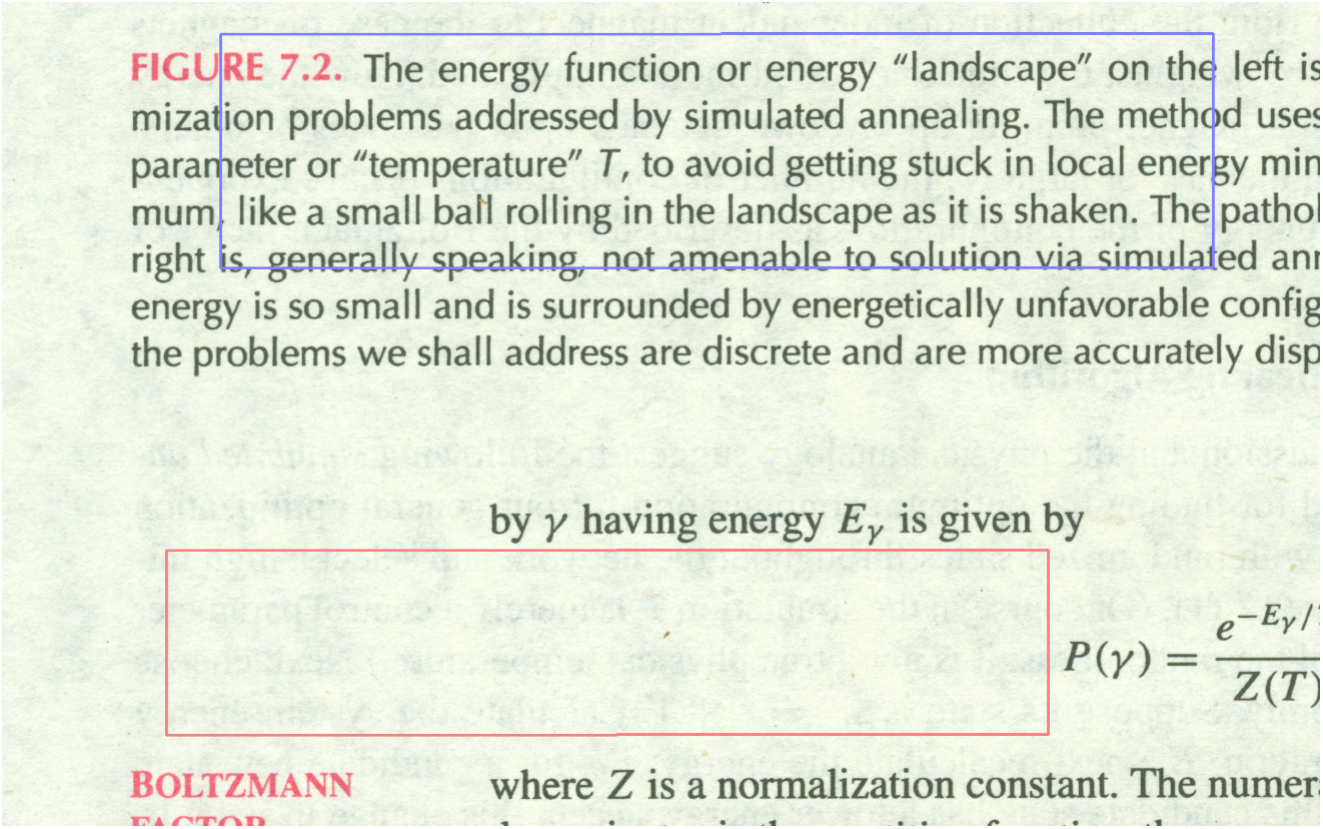
Resulting image obtained by [1]. The red rectangle marks a reverse-side-printed area, and the blue rectangle marks a duplex-printed area.

**Fig 5:**
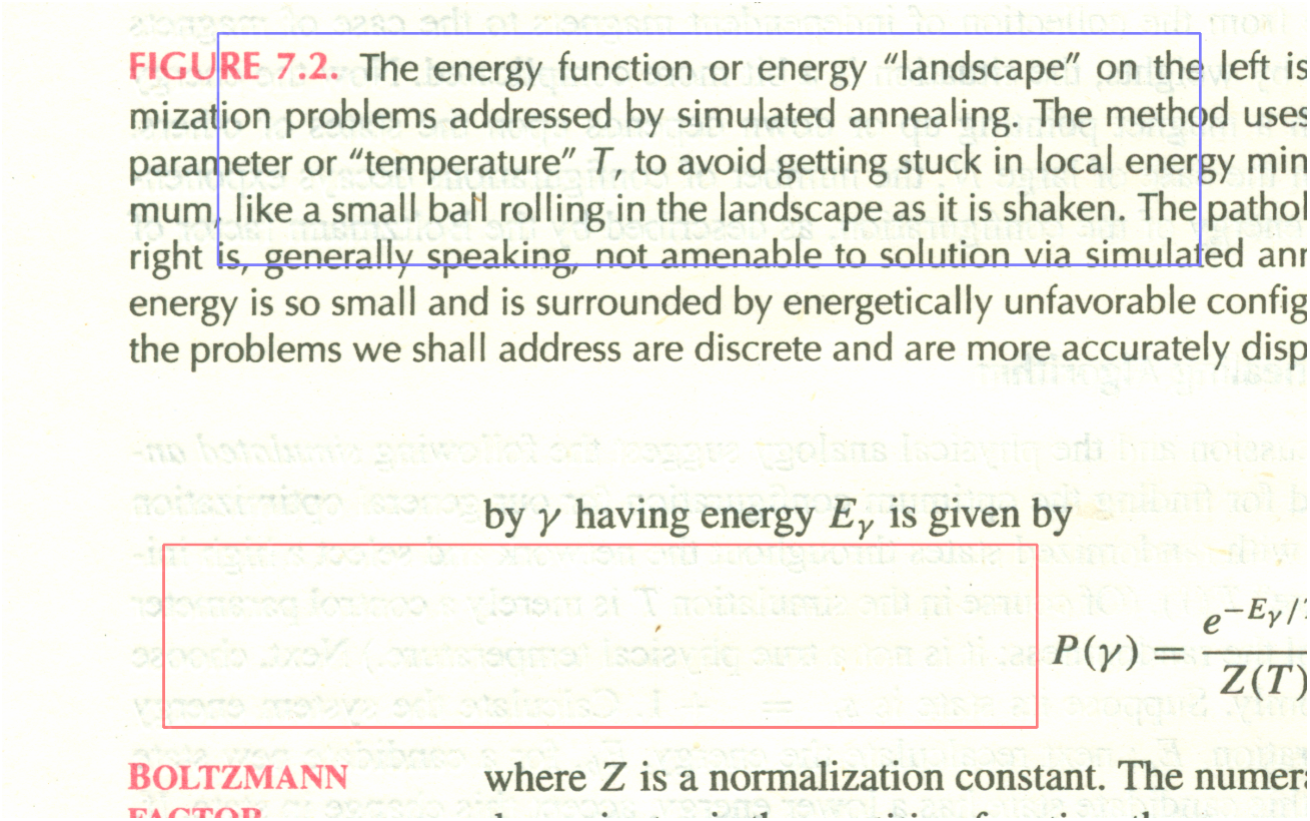
White-backed scan image. The red rectangle marks a reverse-side-printed-only image area, and the blue rectangle marks a duplex-printed area.

A resulting image obtained by the method proposed in [1] is displayed in Fig 4. We can see that the black backing proposed by [1] reduces the show-through back-side printing as compared to the white-backed scan image displayed in Fig 5 thanks to the reduction of the light reflected back from the reverse side. It however brings about material texture of the paper substrate appearing as blueish image texture, which is undesirable.

**Fig 6:**
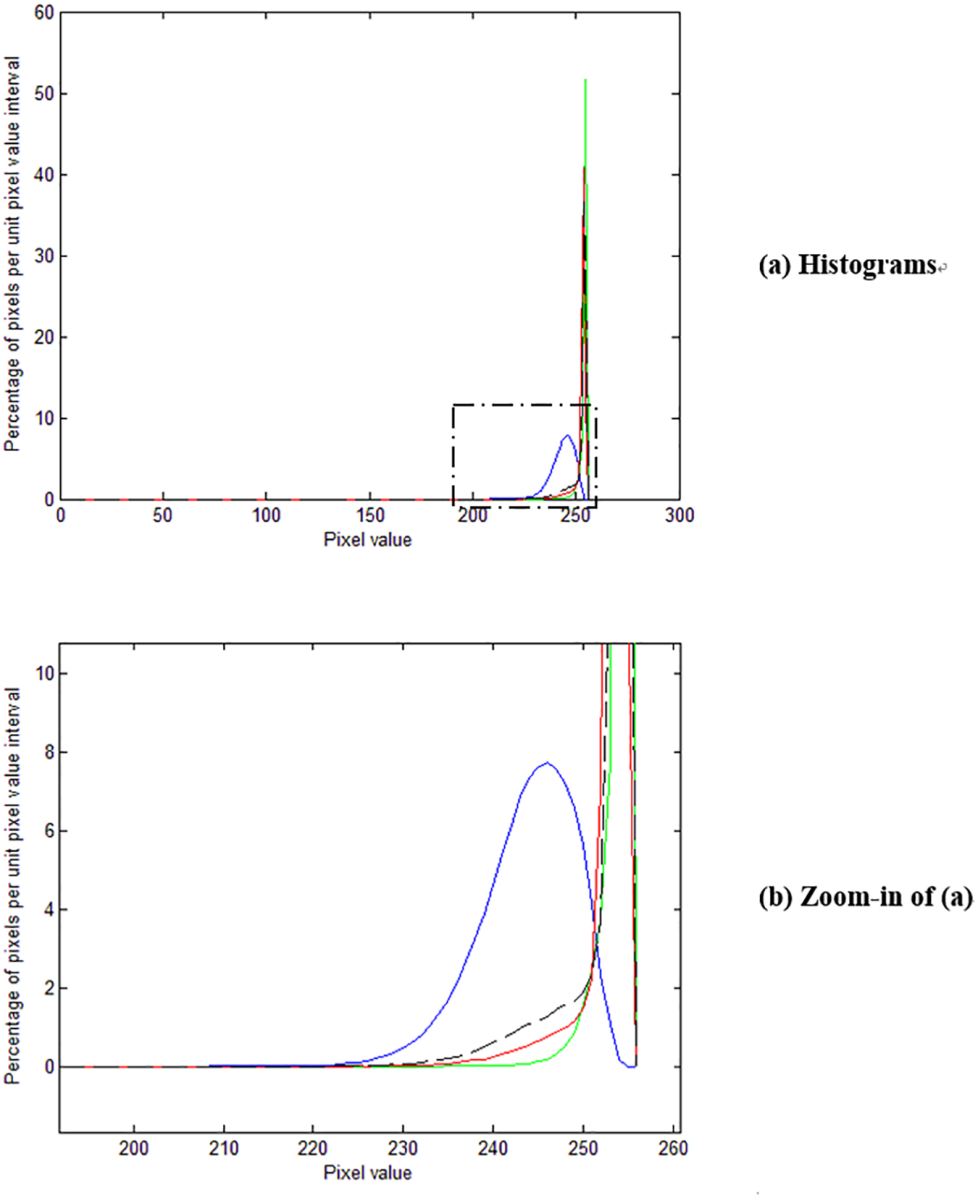
Pixel value histograms of reverse-side-printed area. The blue line is for [1], the red line for [9], the green line for our method, and the dashed black line for the raw white-backed scan image.

We now use the pixel value histogram (the blue line in Fig 6) of a reverse-side-printed area (marked with a red rectangle in Fig 4) to evaluate its performance. We can see from Fig 6 that the histogram is not ideally concentrated at high pixel value location, and in fact the peak appears less concentrated than that for the raw white-backed scan image (the dashed black line in Fig 6). This is explainable since the blueish image texture brought about by [1] increases the dispersion of the peak, and the increase is more than the decrease from the reduction of shown-through reverse-side content.

In order to quantitatively indicate the dispersion of the peak, we use the standard deviation of the histogram. The standard deviation values for [1] are listed in Table 1. We can find from the table that the standard deviation values for [1] are larger than those for raw white-backed scan image. This agrees with our visual inspection of the histogram graphs.

**Table 1:** Standard deviations for each method at each reverse-side-printed area (RSP Area).

	Raw image	Method [1]	Method [9]	Our method
RSP Area #1	4.16	5.51	2.91	1.79
RSP Area #2	3.66	5.15	2.54	1.39
RSP Area #3	3.96	5.28	2.47	1.52
RSP Area #4	4.76	5.32	3.32	1.94
RSP Area #5	4.54	5.17	2.99	1.84
RSP Area #6	4.30	5.02	3.07	1.69

**Fig 7:**
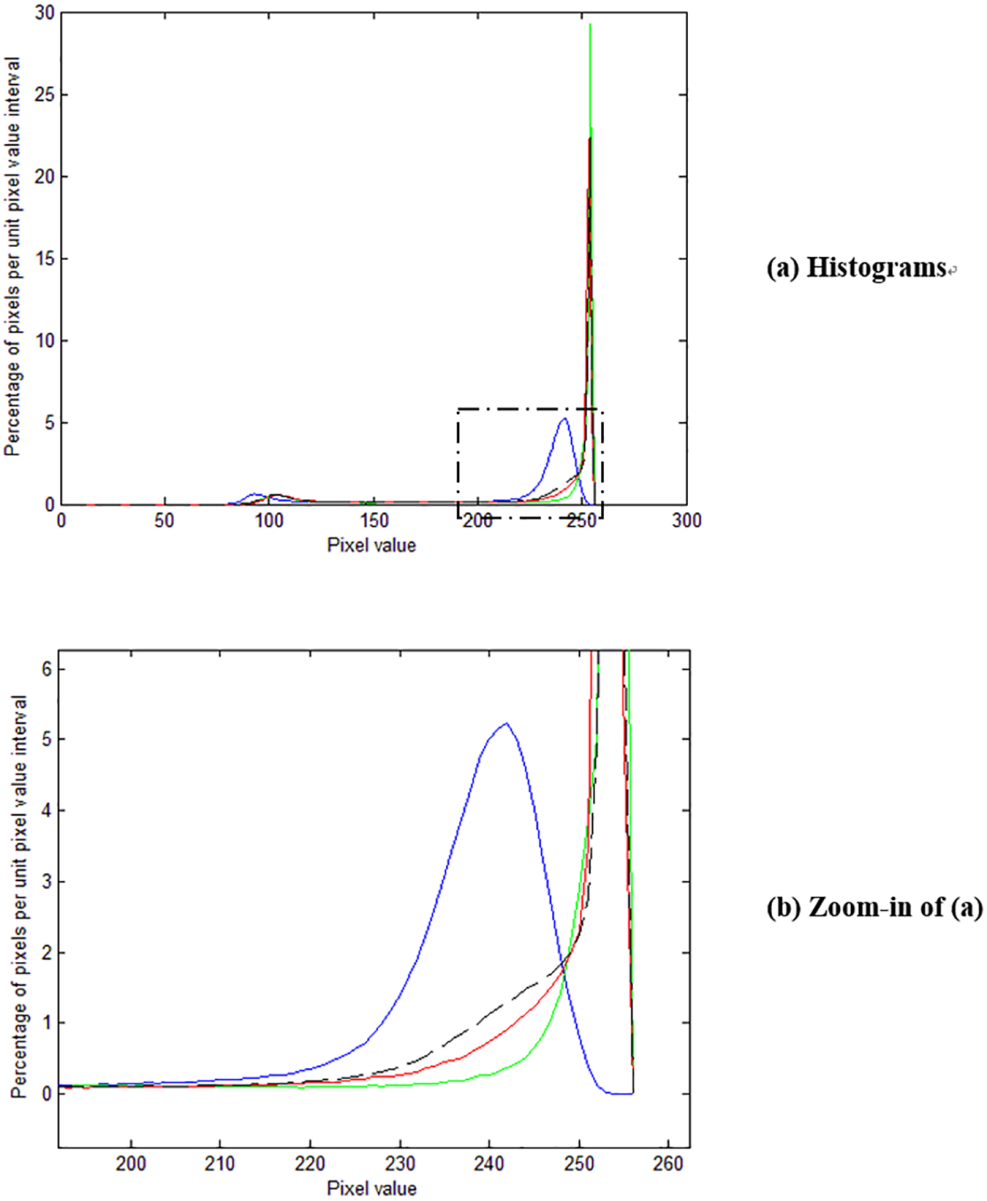
Pixel value histograms of duplex-printed area. The blue line is for [1], the red line for [9], the green line for our method, and the dashed black line for the raw white-backed scan image.

In addition to the pixel value histogram of reverse-side-printed area, we also use the pixel value histogram (the blue line in Fig 7) of a duplex-printed area (marked with a blue rectangle in Fig 4) to analyze its performance. We can see from Fig 7(A) that this histogram has an additional smaller peak to the left of the larger peak. The smaller peak is resulted from the front-side printed text in the area, and the larger peak is resulted from areas containing no front-side content nor reverse-side content. The larger peak also occurs in the pixel value histogram of reverse-side-printed area while the smaller peak does not. We can see from Fig 7(B) that the peak for [1] is not ideally concentrated at high pixel value location, and in fact it appears less concentrated than that for the raw white-backed scan image. This is consistent with the results using the pixel value histogram of reverse-side-printed area.

### Method [9]

**Fig 8:**
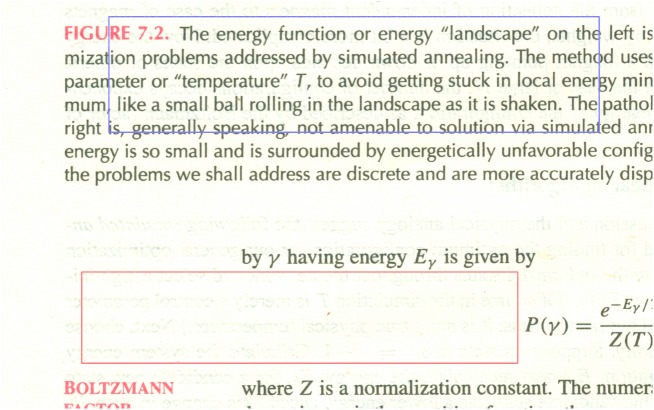
Resulting image obtained by [9]. The red rectangle marks a reverse-side-printed area, and the blue rectangle marks a duplex-printed area.

A resulting image obtained by the method proposed in [9] is displayed in Fig 8. We can see that this method considerably reduces show-through, and moreover, unlike [1], it does not bring about the undesirable material texture of the paper substrate, which is a significant advantage over [1]. We can however still observe visible remnant shown-through reverse-side printing in the resulting image.

We now use the pixel value histogram (the red line in Fig 6) of a reverse-side-printed area (marked with a red rectangle in Fig 8) to analyze its performance. We can see from Fig 6 that the histogram for [9] is more concentrated than that for [1] and that for the raw white-backed scan image but still has some spread over the lower pixel value region (the left side tail), reflecting the visible residue of the leaked-out reverse-side printed text.

We use the standard deviation of the histogram to quantitatively indicate the dispersion of the peak. The standard deviation values for [9] are listed in Table 1. We can find from the table that they are consistently smaller than those for the raw white-backed scan image and those for the resulting image by [1]. This agrees with our visual inspection of the histogram graphs.

In addition to the pixel value histogram of reverse-side-printed area, we also use the pixel value histogram (the red line in Fig 7) of a duplex-printed area (marked with a blue rectangle in Fig 8) to evaluate the performance. We can see from Fig 7(B) that the peak is more concentrated than that for [1] and that for the raw white-backed scan image but still has some spread over the lower pixel value region (the left side tail). This is consistent with the results using the pixel value histogram of reverse-side-printed area.

### Proposed method

**Fig 9:**
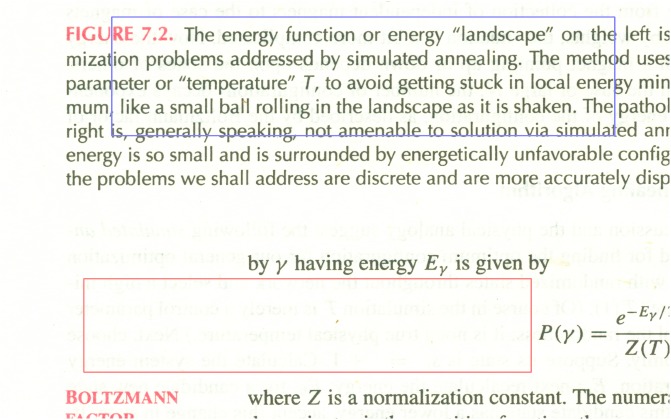
Resulting image obtained by our method. The red rectangle marks a reverse-side-printed area, and the blue rectangle marks a duplex-printed area.

A resulting image obtained by our method is displayed in Fig 9. We can see that it contains significantly less remaining leaked-out reverse-side printing than that of [1] and [9], indicating that our method is more effective than [1] and [9] in removing show-through. Another advantage of our method is that it does not produce unwanted side effects such as bringing about the material texture of the paper substrate.

We now use the pixel value histogram (the green line in Fig 6) of a reverse-side-printed area (marked with a red rectangle in Fig 9) to evaluate its performance. We can see from Fig 6 that the histogram for our method is significantly more concentrated than that for [1] and [9], reflecting its superior show-through elimination ability and the advantage of causing no side effects.

We use the standard deviation of the histogram to quantitatively indicate the dispersion of the peak. The standard deviation values for our method are listed in Table 1. We can find from the table that they are consistently smaller than those for [1] and [9]. This agrees with our visual inspection of the histogram graphs.

In addition to the pixel value histogram of reverse-side-printed area, we also use the pixel value histogram (the green line in Fig 7) of a duplex-printed area (marked with a blue rectangle in Fig 9) to evaluate its performance. We can see from Fig 7(B) that the peak for our method is more concentrated than that for [1] and [9]. This is consistent with the results using the pixel value histogram of reverse-side-printed area.

## Conclusions

We have presented in this paper a novel method for scanning duplex-printed documents without incurring the undesirable show through interference. The main novelty of the proposed method is to achieve the goal of show through free scanning by fusing a white backed scan image with a black backed scan image of the document. The fusion is accomplished using a multilayer perceptron that learns the fusion mapping from manually corrected scan images. We have also proposed to use the pixel value histogram of reverse-side-printed area as well as the pixel value histogram of duplex-printed area to indicate the severity of the show through to enable objective comparison of the methods in consideration. Experiment results show that the proposed method offers substantially stronger show through suppression ability than the two state-of-the-art methods in comparison.

## Supporting information

S1 Fig Scan image of the front side (page 353) with white backing. (BMP)

S2 Fig Scan image of the front side (page 353) with black backing. (BMP)

S3 Fig Scan image of the reverse side (page 354) with white backing. (BMP)

S4 Fig Scan image of the front side (page 493) with white backing. (BMP)

S5 Fig Scan image of the front side (page 493) with black backing. (BMP)

S6 Fig Scan image of the reverse side (page 494) with white backing. (BMP)

## References

[1] Knox KT. Show-through correction for two-sided documents, United States Patent No. 5,832,137 (1998).

[2] Sharma G. Show-through compensation apparatus and method, U.S. Patent Application 09/200984 (1998).

[3] Sharma G. Show-Through Cancellation in Scans of Duplex Printed Documents, IEEE Transactions on Image Processing, Vol. 10, No. 5 (2001). 10.1109/83.918567 18249664

[4] Tonazzini A, Salerno E, Bedini L. Fast correction of bleedthrough distortion in grayscale documents by a Blind Source Separation technique, International Journal on Document Analysis and Recognition, Vol. 10, pp. 17–25 (2007).

[5] Tonazzini A, Bianco G, Salerno E. Registration and enhancement of double-sided degraded manuscripts acquired in multispectral modality, in 10th International Conference on Document Analysis and Recognition (2009).

[6] Bayat FM, Zadeh MB, Jutten C. Using non-negative matrix factorization for removing showthrough, in Proc. LVA/ICA, pp. 482–489 (2010).

[7] Bayat FM, Zadeh MB, Jutten C. Linearquadratic blind source separating structure for removing show-through in scanned documents, Int. J. on Document Anal. and Recogn., vol. 14, pp. 319–333 (2011).

[8] Fan Z, Eschbach R, Maltz MS, Stinehour J. Show-through reduction method and system, United States Patent No. 7,965,421 (2011).

[9] Liu Q, Wang W. Show-through removal for scanned images using non-linear NMF with adaptive smoothing, in IEEE China Summit and International Conference on Signal and Information Processing (2013). 10.1109/ChinaSIP.2013.6625340 PMC5382957 28393144

[10] Lowe DG. Distinctive image features from scale-invariant key points, International Journal of Computer Vision 60(2): 91–110 (2004).

[11] Wu J, Cui Z, Sheng VS, Zhao P, Su D, Gong S. A Comparative Study of SIFT and its Variants, Measurement Science Review. Volume 13, Issue 3, Pages 122–131 (2013).

[12] Bay H, Tuytelaars T, Van Gool L. Surf: Speeded up robust features, in European Conference on Computer Vision, pages 404–417, (2006).

[13] Smith SM, Brady JM. SUSAN–a new approach to low level image processing, International Journal of Computer Vision 23 (1): 45–78 (1997).

[14] Fischler MA, Bolles RC. Random sample consensus: a paradigm for model fitting with applications to image analysis and automated cartography, Communications of the ACM, Volume 24, Issue 6, Pages 381–395 (1981).

[15] Raguram R, Frahm JM, Pollefeys M. A Comparative Analysis of RANSAC Techniques Leading to Adaptive Real-Time Random Sample Consensus, in European Conference on Computer Vision (2008).

[16] Rumelhart DE, Hinton GE, Williams RJ. Learning representations by back-propagating errors, Nature 323, Pages 533–536 (1986).

[17] Møller MF. A scaled conjugate gradient algorithm for fast supervised learning, Neural Networks, Volume 6, Issue 4, Pages 525–533 (1993).

[18] Open Source Computer Vision (OpenCV), Available at: http://opencv.org/.

[19] Fast Neural Network Library (FANN), Available at: http://leenissen.dk/fann/wp/.

