# Supplementary figures and images for: Scanning double-sided documents without incurring show-through by learning to fuse two complementary images using multilayer perceptron
Source: PLoS One. 2017 May 10;12(5):e0176969. doi: 10.1371/journal.pone.0176969 (PMC5425205; doi:10.1371/journal.pone.0176969)

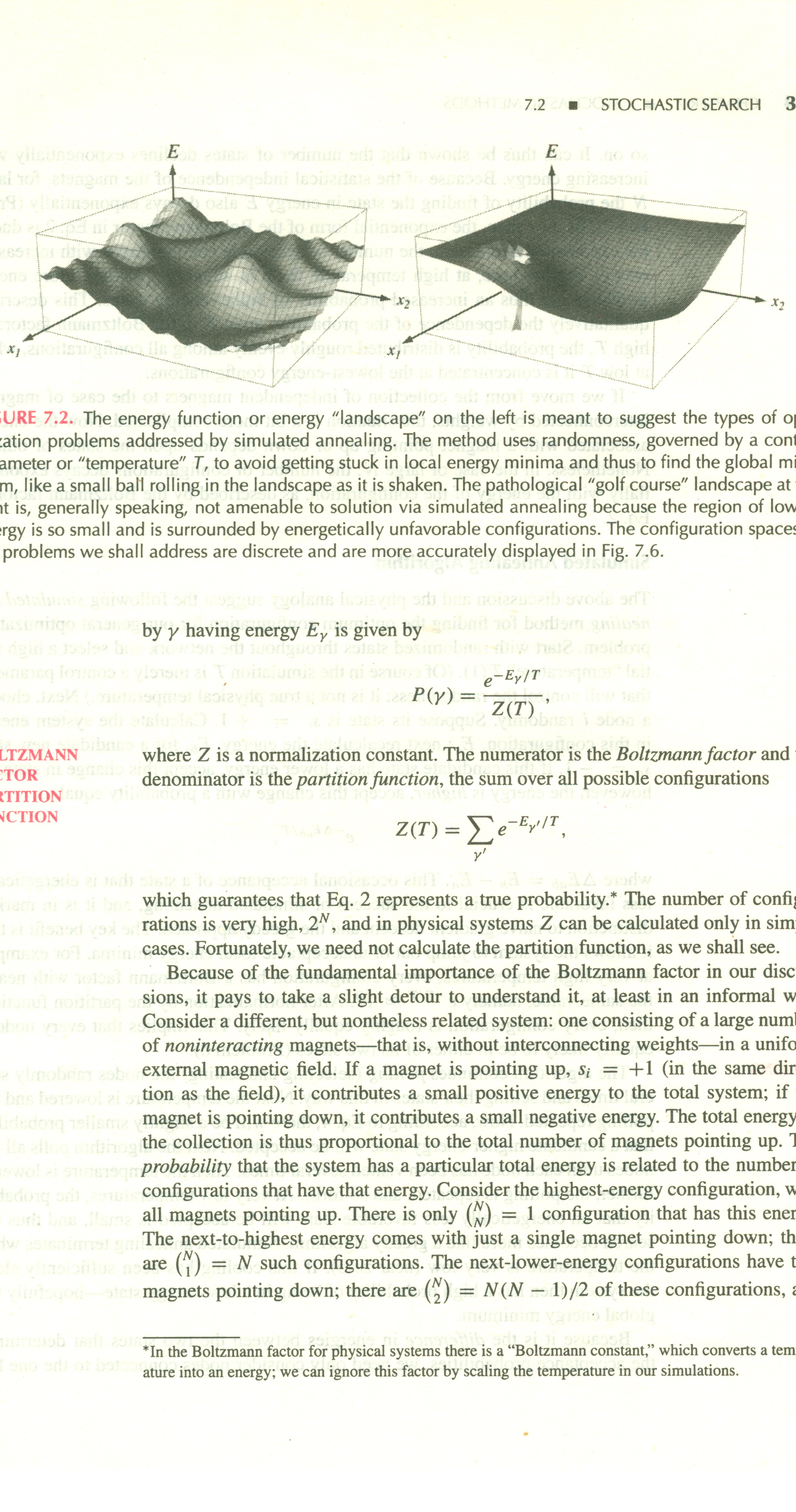

Supplement: S1 Fig — (BMP) [file pone.0176969.s001.bmp]

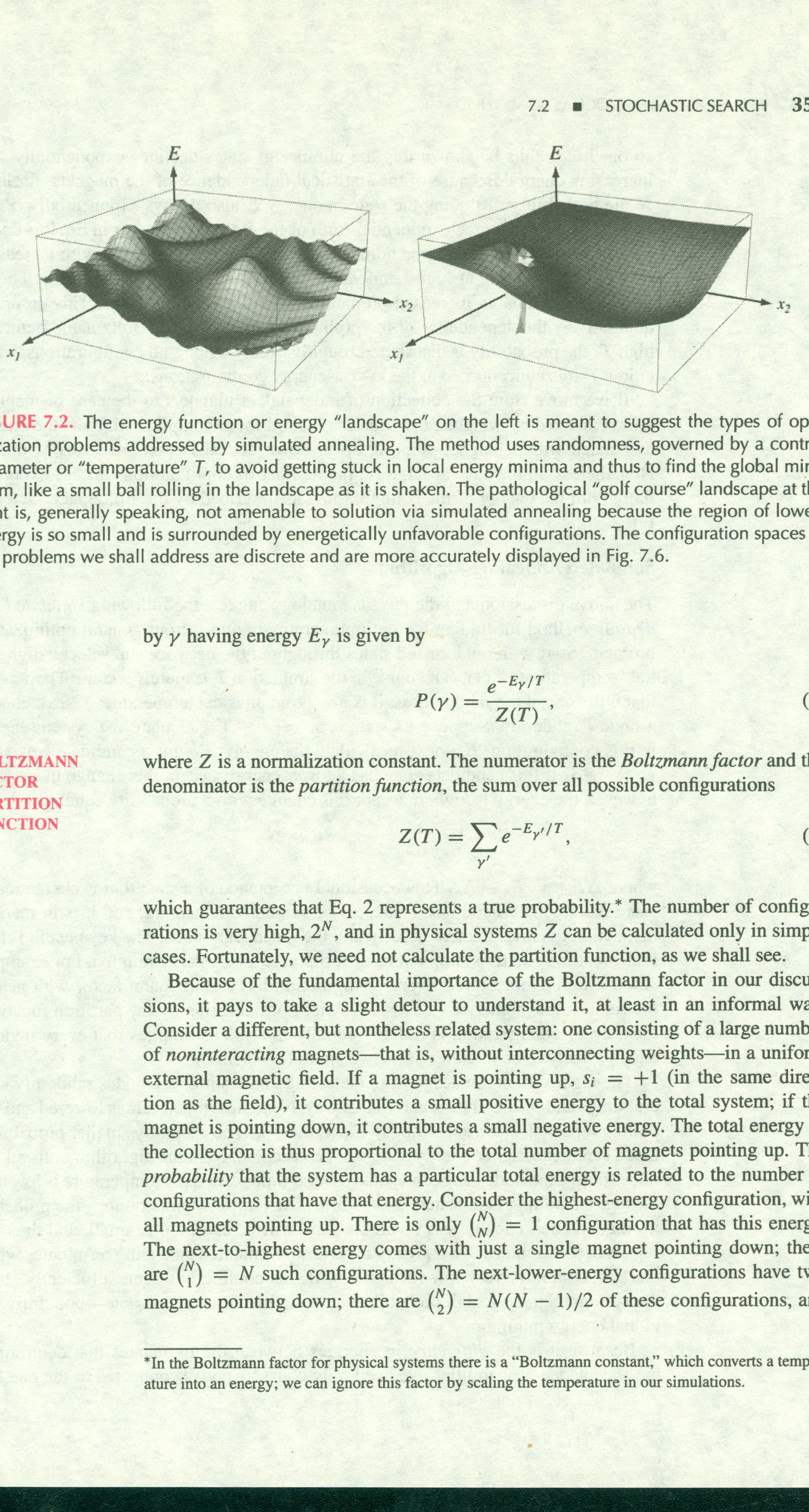

Supplement: S2 Fig — (BMP) [file pone.0176969.s002.bmp]

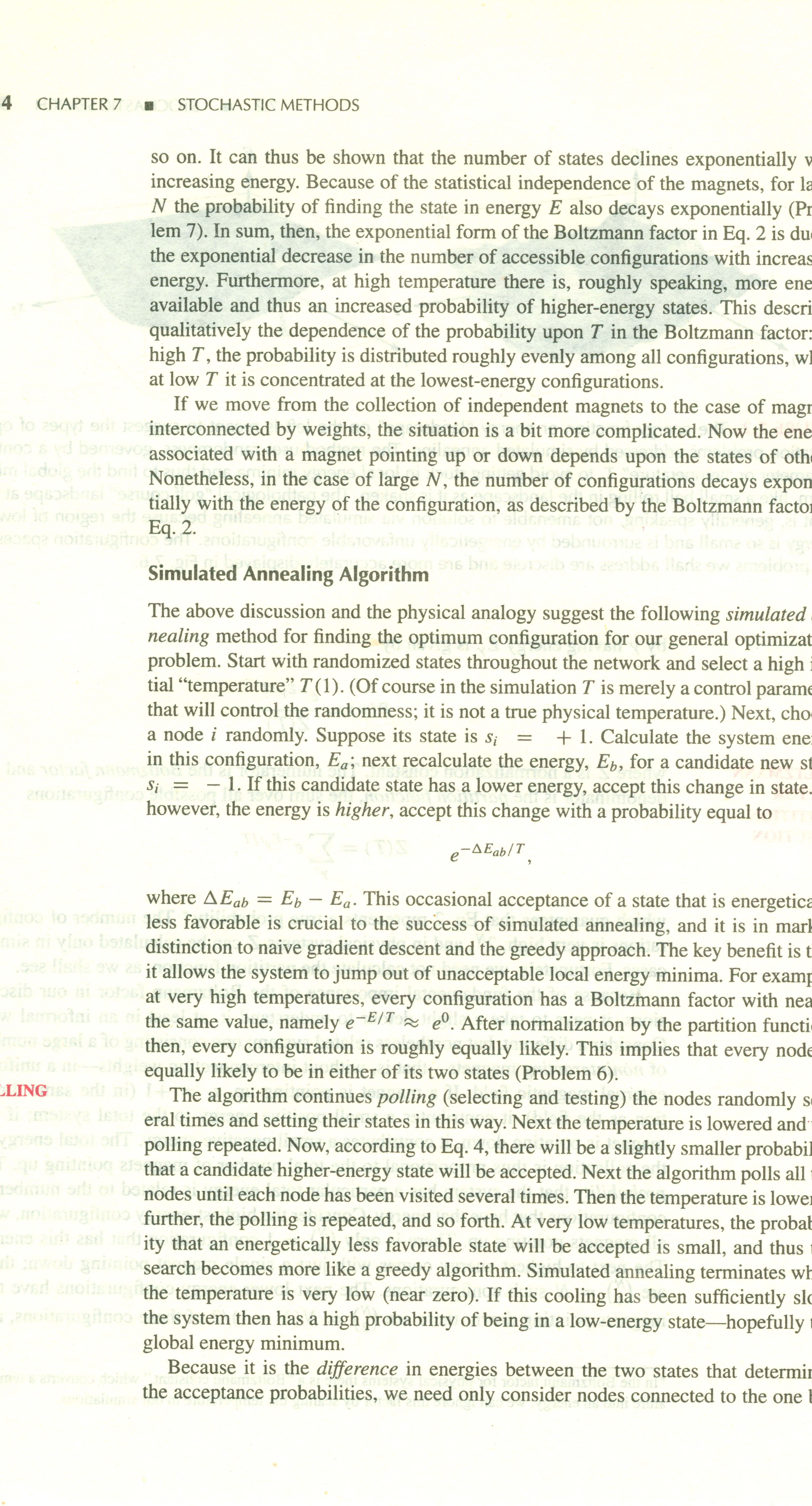

Supplement: S3 Fig — (BMP) [file pone.0176969.s003.bmp]

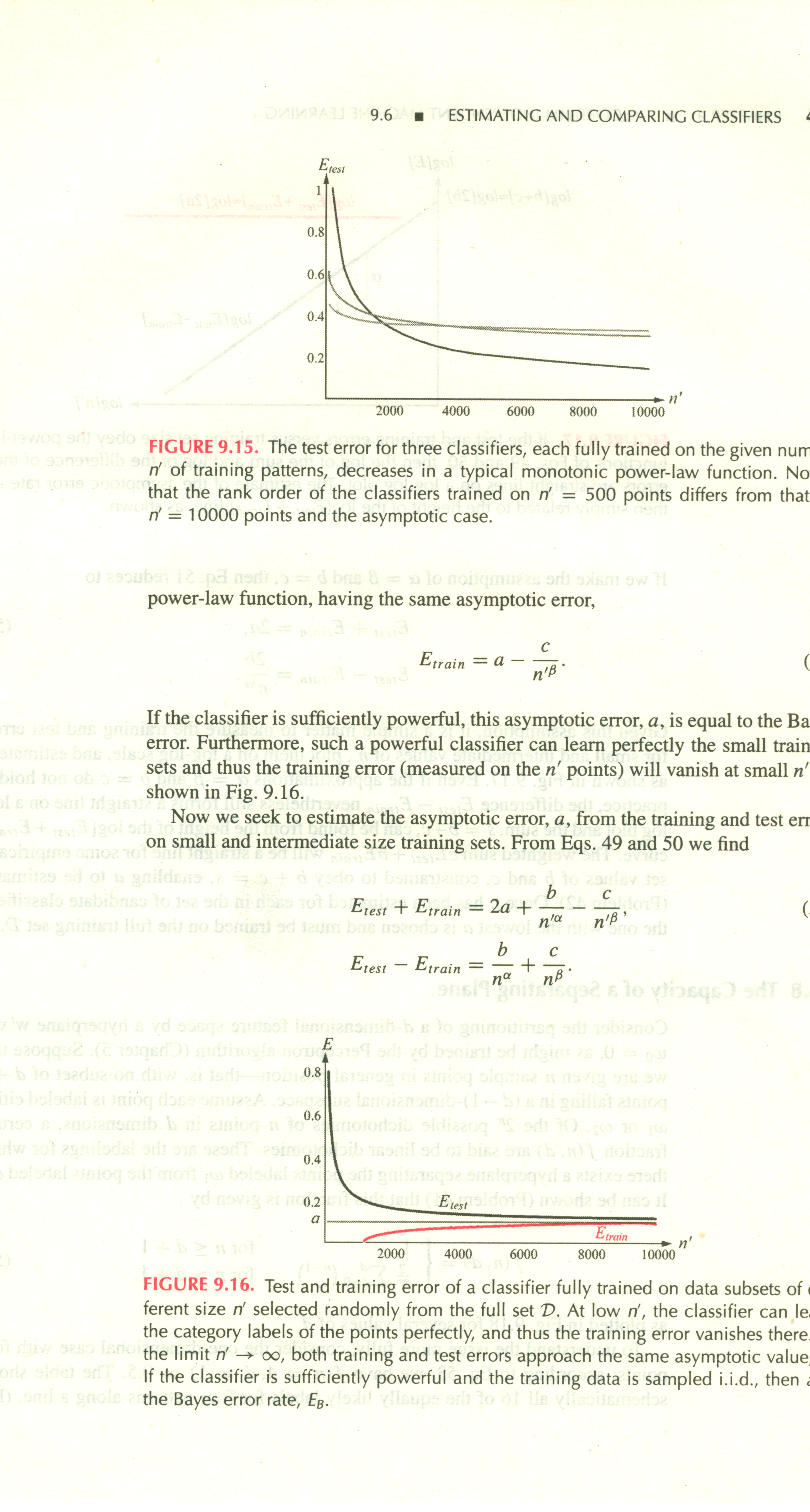

Supplement: S4 Fig — (BMP) [file pone.0176969.s004.bmp]

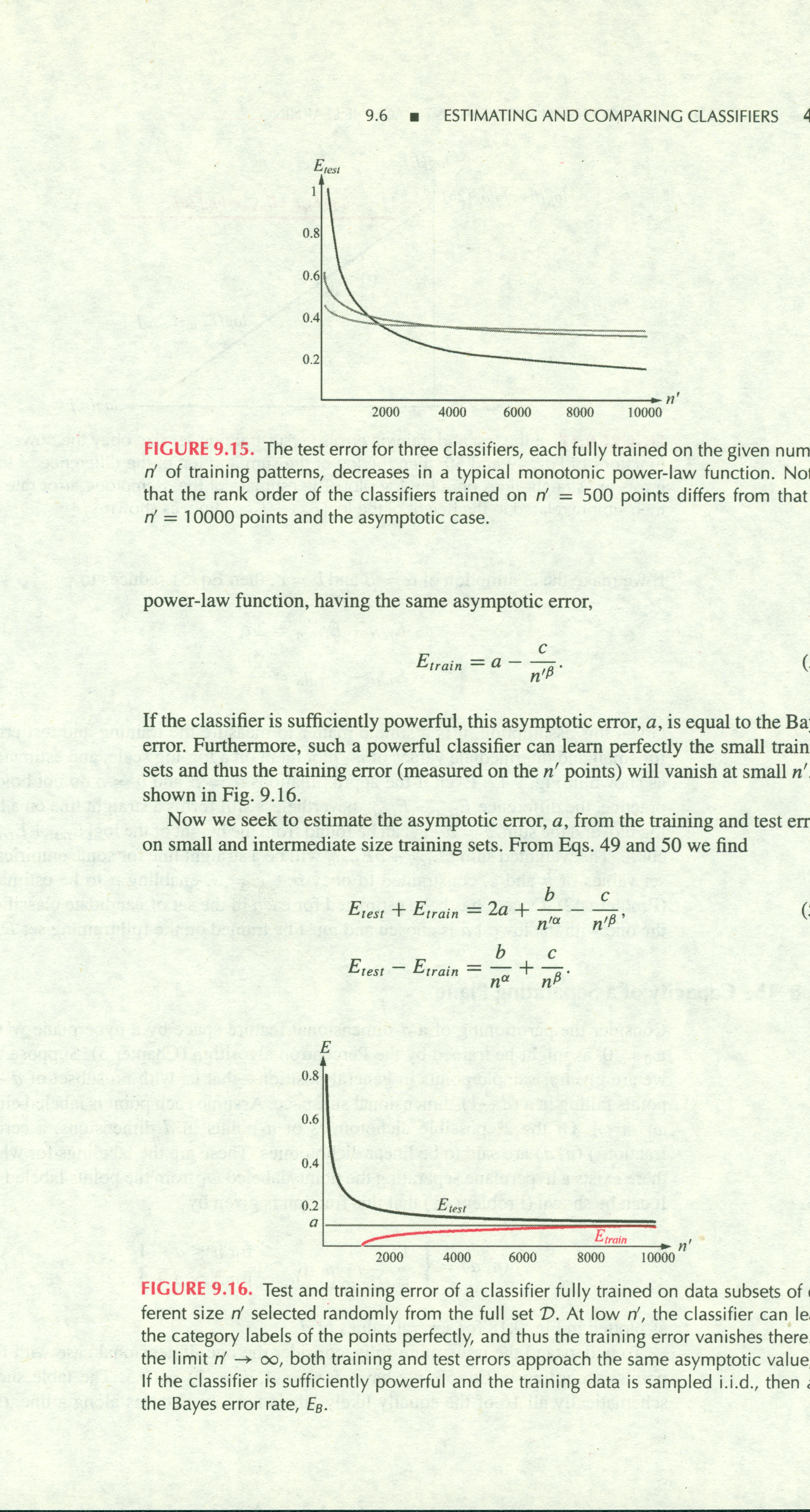

Supplement: S5 Fig — (BMP) [file pone.0176969.s005.bmp]

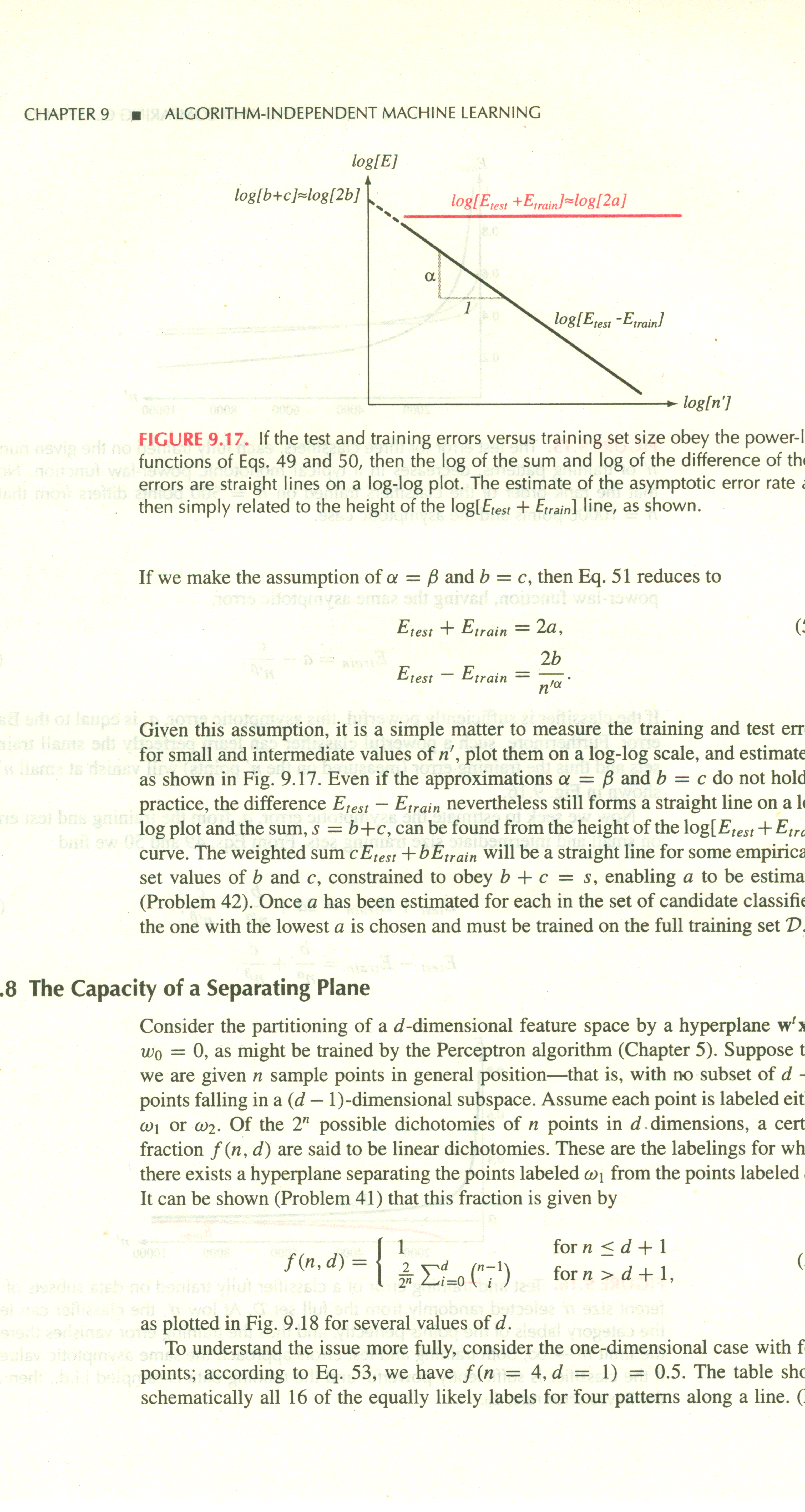

Supplement: S6 Fig — (BMP) [file pone.0176969.s006.bmp]
